# Superior and Inferior Ophthalmic Vein Thrombosis in the Setting of Lung Cancer

**DOI:** 10.1155/2018/6025274

**Published:** 2018-10-15

**Authors:** Nawal Habib, Kimberly Lessard

**Affiliations:** Department of Internal Medicine, Einstein Medical Center Philadelphia, Philadelphia, USA

## Abstract

Superior ophthalmic vein thrombosis is extremely rare and is often associated with orbital inflammation/infection, systemic/local tumors, hypercoagulable states, autoimmune conditions, and rarely carotid cavernous fistula. Clinical features include abrupt onset of painful proptosis, chemosis, ophthalmoplegia, and diminished visual acuity. Prompt diagnosis and treatment are essential to prevent permanent blindness. Management options for this medical emergency include antibiotics, steroids, and/or anticoagulation. We present a case of superior ophthalmic vein thrombosis with left cavernous sinus thrombosis in the setting of newly diagnosed malignancy.

## 1. Introduction

Superior ophthalmic vein thrombosis is a rare diagnosis that is generally associated with infectious conditions and hypercoagulable states related to cancer and/or autoimmune conditions. Although there are limited management guidelines available for this medical emergency, prompt diagnosis and treatment are essential to prevent permanent blindness. We present a case of superior ophthalmic vein thrombosis with left cavernous sinus thrombosis in the setting of newly diagnosed malignancy.

## 2. Case Presentation

**Figure 1:**
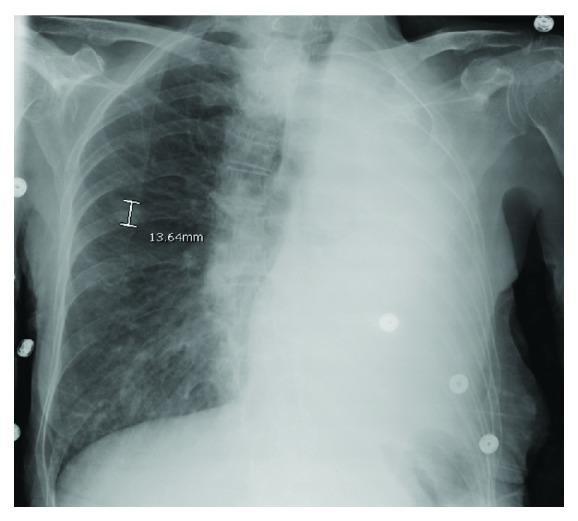


**Figure 2:**
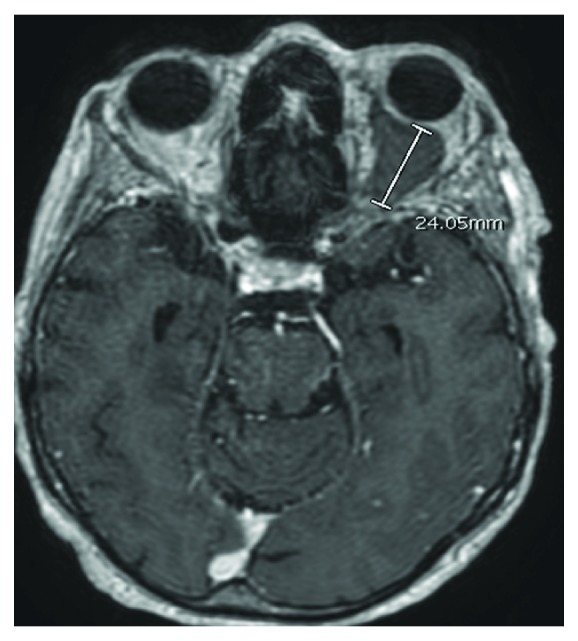


**Figure 3:**
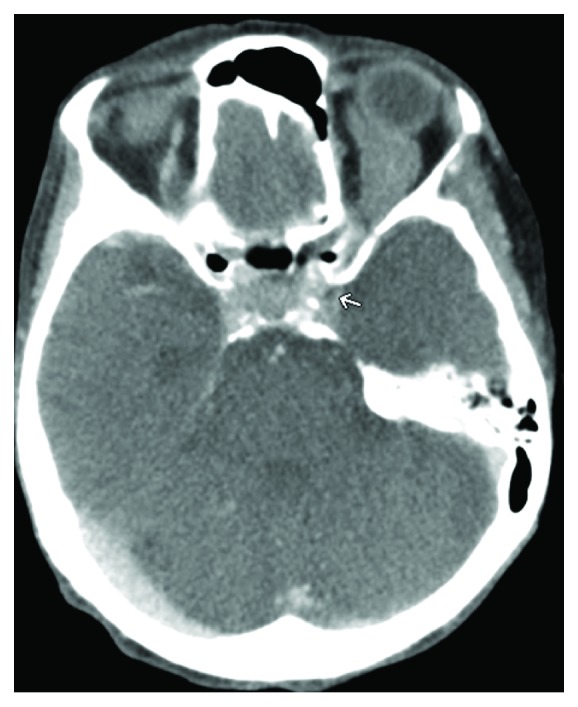


An 82-year-old female with a past medical history of uncontrolled hypertension, dementia, and tobacco abuse presented with complaints of blurry vision and frequent falls for the past few weeks. She also noted recent unintentional weight loss and productive cough. She was afebrile, cachectic, had decreased left-sided breath sounds, mild left-sided proptosis, chemosis, and bilateral cataracts on admission. Ophthalmology was consulted and reported that visual acuity was R 20/25 and L hand motion, with normal pupillary function and extraocular movements. Cranial nerve functions were grossly intact and intraocular pressures were within normal limits. Lab results and initial CT head were within normal limits. Chest X-ray (Figure 1) and CT alike showed left-sided atelectasis with ipsilateral mediastinal shift due to mucus plugging, a moderate left pleural effusion, and multiple spiculated cavitating nodules in the right lobe highly suspicious for malignancy. Two days following admission, she developed acute left orbital pain and visual loss prompting a brain MRI. MRI revealed enlargement of the L superior and inferior ophthalmic veins without contrast enhancement concerning for thrombosis (Figure 2). Following initiation of heparin infusion, a CT venogram (CTV) confirmed the presence of thrombosis along with a filling defect in the L cavernous sinus (Figure 3). CTV is felt to be the best diagnostic modality for detecting venous thrombosis.

In the absence of signs, symptoms, or risk factors for underlying infection, newly diagnosed malignancy remained the primary and most likely differential, and antibiotics were not initiated. Unfortunately, several barriers to confirmation of malignancy arose. Bronchoscopy was performed which, despite removal of a mucus plug, resulted in minimal improvement in the atelectasis. Pathology from brush cytology and transbronchial biopsy were inconclusive. A transthoracic lung biopsy of right lung nodules was deemed a high risk given persistent atelectasis of the left lung and presence of underlying emphysema. A thoracentesis of the left effusion was performed; however, cytology was negative for malignancy. Given the negative autoimmune and relevant hematologic work-up for hypercoagulability and the high likelihood of newly diagnosed underlying lung cancer, she was started on Lovenox with subsequent improvement in visual symptoms over the next few weeks. Serial chest CT will be required to monitor for progression.

## 3. Discussion

Risk factors for superior ophthalmic vein thrombosis (SOVT) are multifactorial and include sinoorbital infections, autoimmune disorders (such as systemic lupus erythematosus, sarcoidosis, Behcet's syndrome, and Grave's disease), hypercoagulable states (such as pregnancy, use of oral contraceptive pills, sickle cell trait, and hereditary hemorrhagic telangiectasia), and systemic malignancies. The elements comprising Virchow's triad (vascular stasis, endothelial damage, and hypercoagulability) play a vital role in the pathophysiology [1]. Clinical features include abrupt onset of painful proptosis, chemosis, ophthalmoplegia, and diminished visual acuity. Isolated SOVT is extremely rare, and the association of SOVT and cavernous sinus thrombosis is more commonly seen [2]. Urgent MR venogram (MRV)/CTV are required to confirm the diagnosis and exclude both sinoorbital infection and potentially fatal cavernous sinus thrombosis. MRV is the preferred modality given the higher sensitivity and ability to detect the patency of the cavernous sinuses with less artifacts compared to the CTV [3]. Diagnostic findings include dilation of the superior ophthalmic vein without contrast enhancement indicating thrombosis and diffusely engorged extraocular muscles due to venous congestion. The relevant hematological and autoimmune work-up includes testing for ANA, ANCA, anti-ds-DNA, anti-Sm, antiphospholipid antibodies, anticardiolipin antibodies, protein C, protein S, antithrombin III, and homocysteine.

There are limited management guidelines for this medical emergency. The severity of symptoms and underlying systemic condition guide prompt management to prevent permanent blindness. Systemic antibiotics are beneficial in cases related to infections. Steroids may be used to relieve orbital inflammation and congestion and thus decrease proptosis [4]. The use of anticoagulation for malignancy-associated cases is to be determined after risk-benefit analysis.

## Additional Points

*Learning Points*. (i) Superior ophthalmic vein thrombosis is extremely rare and is often associated with orbital inflammation/infection, systemic/local tumors, hypercoagulable states, and autoimmune conditions. (ii) Clinical features include abrupt onset of painful proptosis, chemosis, ophthalmoplegia, and diminished visual acuity. (iii) Urgent MR venogram (MRV)/CTV are required to confirm the diagnosis and exclude both sinoorbital infection and potentially fatal cavernous sinus thrombosis. (iv) Management options for this medical emergency include antibiotics, steroids, and/or anticoagulation.
